# Generating Concentration Gradients across Membranes for Molecular Dynamics Simulations of Periodic Systems

**DOI:** 10.3390/ijms25073616

**Published:** 2024-03-23

**Authors:** Eric Joon Shinn, Emad Tajkhorshid

**Affiliations:** Theoretical and Computational Biophysics Group, NIH Resource Center for Macromolecular Modeling and Visualization, Beckman Institute for Advanced Science and Technology, Department of Biochemistry, Center for Biophysics and Quantitative Biology, University of Illinois at Urbana-Champaign, Urbana, IL 61801, USA; shinn3@illinois.edu

**Keywords:** membranes, lipid bilayers, molecular dynamics, oxygen permeability, gradient simulations

## Abstract

The plasma membrane forms the boundary between a living entity and its environment and acts as a barrier to permeation and flow of substances. Several computational means of calculating permeability have been implemented for molecular dynamics (MD) simulations-based approaches. Except for double bilayer systems, most permeability studies have been performed under equilibrium conditions, in large part due to the challenges associated with creating concentration gradients in simulations utilizing periodic boundary conditions. To enhance the scientific understanding of permeation and complement the existing computational means of characterizing membrane permeability, we developed a non-equilibrium method that enables the generation and maintenance of steady-state gradients in MD simulations. We utilize PBCs advantageously by imposing a directional bias to the motion of permeants so that their crossing of the boundary replenishes the gradient, like a previous study on ions. Under these conditions, a net flow of permeants across membranes may be observed to determine bulk permeability by a direct application of J=PΔc. In the present study, we explore the results of its application to an exemplary $O_{2}$ and POPC bilayer system, demonstrating accurate and precise permeability measurements. In addition, we illustrate the impact of permeant concentration and the choice of thermostat on the permeability. Moreover, we demonstrate that energetics of permeation can be closely examined by the dissipation of the gradient across the membrane to gain nuanced insights into the thermodynamics of permeability.

## 1. Introduction

At the cellular level, the boundary between a living entity and its environment is the plasma membrane. Composed of lipids [1], proteins, and glycans, the plasma membrane acts as a barrier to permeation and enables the regulated flow of metabolites in homeostasis [2]. Substances may traverse the membrane in several ways. They may be transported via the vesicle-mediated endo- and exocytosis, which involve topological transformations of the membrane [3]. Substrates may also cross membranes facilitated by integral membrane proteins, either by passive diffusion through a channel, or by active transfer by a transporter involving expenditure of energy [4,5]. ABC (ATP Binding Cassette) transporters, for example, which constitute the largest class of transporters, use ATP binding and hydrolysis to actively transport ions or other molecules in a particular direction (import or export) across the membrane [6]. As an example of passive channels, aquaporins have been shown to substantially increase the permeability of cell membranes to water [7,8]. Finally, molecules can cross across membranes passively by just dissolving into the lipid phase and back out into the solvent on the other side. Small, non-polar or amphiphilic molecules are believed to traverse the membrane primarily by this mechanism [9].

### 1.1. Existing Computational Methods for Characterizing Membrane Permeability

Much scientific effort has been dedicated to characterizing permeability [10,11,12,13]. Under steady-state conditions, the permeability *P* of a boundary to a solute is described by the equation [11,12,13,14],

(1) J=PΔc,

where *J* is the rate of permeant flux across the boundary per unit area, and Δc is the difference in permeant concentration across the boundary. This description of permeability is derived from Fick’s first law of diffusion [15,16].

Several computational means of calculating permeability have been implemented for molecular dynamics (MD) simulations. One approach uses the solubility-diffusion model, which originates from Overton’s rule [9,17,18], to predict the permeability of a membrane from the solubility and diffusivity of a permeant [19,20,21]. The inhomogeneous solubility-diffusion (ISD) [11,22,23,24] model extends the idea by decomposing the resistance to permeation, $R_{p}$, posed by, for example, a membrane into infinitesimal contributions:

(2) $$R_{p}=\frac{1}{P}=c^{*}\int_{-L/2}^{+L/2}\frac{dz}{c^{\;eq}\left(z\right)D\left(z\right)}=\int_{-L/2}^{+L/2}\frac{dz}{e^{-w\left(z\right)/k_{B}T}D\left(z\right)}$$

where $c^{*}$ is the concentration of the permeant in solution, $c^{eq}\left(z\right)$ is the concentration of the permeant at position *z* within the membrane, and *w* and *D* are the potential of mean force (PMF) and diffusivity of the permeant at *z*, respectively, with the range between ±L/2 encompassing the width of the membrane. Many computationally derived predictions of permeability have used the ISD model to yield reasonable estimates for a wide variety of membranes and permeants [22,23,25,26,27,28,29,30,31].

Alternatively, counting-based approaches aim to predict membrane permeability in a more consistent manner to the experiments and the theory, by observing the rate of permeation events across a membrane to calculate permeability by a direct application of J=PΔc (Equation (1)). This approach requires minimal theoretical assumptions and has been applied to study various systems, recently for $H_{2}$O, $O_{2}$, and ethanol permeation across POPC bilayers [12,31].

Despite their success, these methods are not without their shortcomings, perhaps the most important being the absence of a concentration gradient, which is the way measurements are taken in experiments. With the exception of double bilayer systems, the majority of simulation permeability studies have been performed under equilibrium conditions, in large part due to the challenges associated with creating concentration gradients in simulations utilizing periodic boundary conditions (PBCs). The gradient is a crucial feature since the presence of a permeant can modulate the properties of the membrane [31], even impacting each leaflet of the lipid bilayer differently. In particular, for approaches using the ISD model, calculations are commonly performed for a single permeation path, and as a result, if the membrane is heterogeneous, even extensive sampling may not guarantee that the variability in local solubilities and diffusivities will be fully represented. Moreover, complexity arising from the permeant itself can add further degrees of freedom (i.e., rotational and conformational), evident, for example, in a recent investigation of chloroquine [32].

Because the ISD model attempts to detail the permeation process by granularizing the properties of the membrane and states of the permeant, the permeation path can become prohibitively complex, not to mention the theoretical assumptions underlying the model such as that of “small gradients” [23].

### 1.2. Need for Generating Concentration Gradients in MD Simulations

To enable studies of permeability under concentration gradient conditions and complement the existing computational means, we developed a non-equilibrium method that enables the generation and maintenance of steady-state gradients in MD simulations. Similar to a previous study [33], we aim to create a concentration gradient across the membrane. In our method, however, we utilize PBCs advantageously to impose directional bias to the motion of permeants so that their crossing at the boundary replenishes the gradient. Under these conditions, a net flow of permeants across membranes may be observed to determine bulk permeability by a direct application of J=PΔc (Equation (1)).

In the present study, we explore the results of the method’s application to an exemplary $O_{2}$ and POPC bilayer system, demonstrating accurate and precise permeability measurements under conditions typical of MD simulations. Because standard practice in MD commonly involves some form of artificiality to acquire adequate sampling for statistics, in our case, unphysiologically high concentrations of $O_{2}$, special care must be taken to make nuanced interpretations of results. We illustrate the impact on permeability made by the permeant concentration choice of thermostat in simulation. Moreover, we demonstrate that energetics of permeation can be closely examined by the dissipation of the gradient across the membrane to gain nuanced insights into the thermodynamics of permeability.

## 2. Results and Discussion

### 2.1. Generating Concentration Gradient in a Water Box

**Figure 1:**
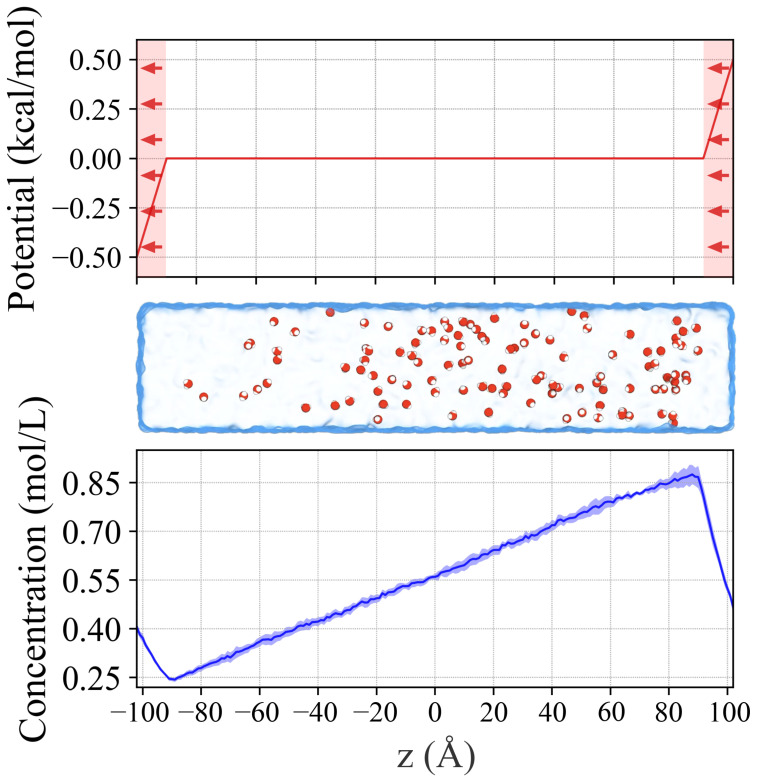
To demonstration the concentration gradient method with water a unidirectional bias was applied to one out of every hundred water molecules in a pure water simulation. The grid potential (top plot) applies a bias only in a small region near the periodic boundaries along the *z*-axis (membrane normal), creating a force that pushes the selected water molecules across the boundary in one direction. In the diagram, the direction of the bias is right to left. The potential is flat within the majority of the simulation system (−90 Å <z< +90 Å) allowing unbiased motion of the particles. The fixed 1 kcal/mol energy ramp between periodic images specified using the G-SMD module in NAMD creates a force on the select particles in the direction indicated by the red arrows. The average concentration of the select water molecules, profiled along the z-axis (bottom plot), displayed a linear gradient within the unbiased region. The blue line shows the average concentration, with the shaded regions indicating the bounds within 1 standard error of the mean.

In a demonstrative application of our technique, one out of every hundred molecules in a pure water box were randomly selected to be subjected to the directional bias, which imposed a 1 kcal/mol energy ramp across the periodic boundary (Figure 1). The measurement of the local concentrations of these water molecules, averaged over the course of a 600 ns simulation, revealed a linear gradient, indicating a steady state was achieved by the method (Figure 1). Moreover, the ratio of the local concentrations of the selected water at the ends of the unbiased region reflected the 1 kcal/mol energy step between periodic images ($\beta \ln\left(\frac{\left[H_{2}O\right]\left(z=+90\;Å\right)}{\left[H_{2}O\right]\left(z=-90\;Å\right)}\right)=\beta \ln\left(\frac{0.868}{0.245}\right)=0.779\;\mathrm{kcal}/\mathrm{mol}\approx 1\;\mathrm{kcal}/\mathrm{mol}$). Some loss in energy is experienced as reflected by the difference in concentration due to dissipation by flow of the selected water molecules. By modulating the magnitude of the energy ramp, users can predictably tune the target concentration gradient to suit their needs.

### 2.2. Generating Oxygen Gradient across a Lipid Bilayer

As a second practical test for our concentration gradient method, gradients of $O_{2}$ were generated across a POPC bilayer for the study of membrane permeability. The $O_{2}$-POPC system was selected as a test case due to the opportunity for abundant sampling provided by the rapid permeation of the small $O_{2}$ molecules, as well as being an exemplary system, well examined in prior studies [29,31,34].

**Figure 2:**
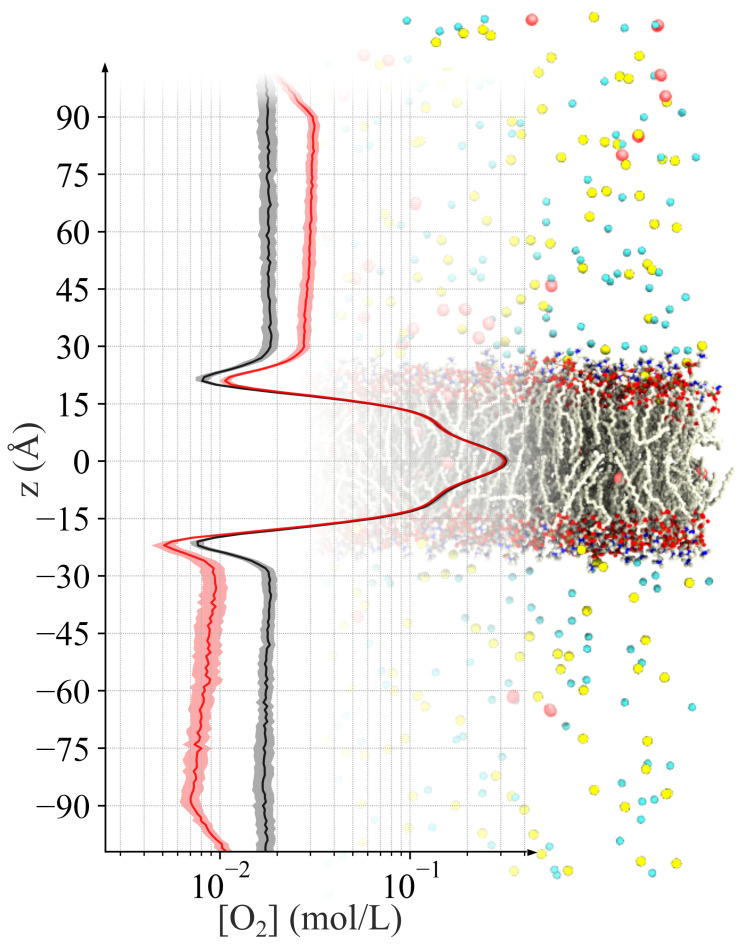
Concentration gradients were generated across simulated lipid bilayers in an application of our method. The molecular image on the right depicts a POPC bilayer centered at z=0 with a ball-and-stick representation in which lipid tails are colored white, while oxygen and nitrogen atoms of the head groups are colored red and blue, respectively. On either side of the bilayer is the aqueous phase which contains ${\mathrm{Na}}^{+}$ (yellow) and ${\mathrm{Cl}}^{-}$ ions colored yellow and cyan, respectively. Water molecules are not shown for clarity. Molecular oxygen ($O_{2}$), shown as two joined red spheres, is present throughout the systems. Imposing a 1 kcal/mol energy step between periodic images results in the enrichment of $O_{2}$ molecules on one side of the membrane while depleting from the other (red plot). A control simulation with no bias results in equal solution concentrations of $O_{2}$ on the two sides of the membrane (black plot). In both cases, the amphiphilic nature of the bilayer is evident; the polar headgroups coincide with a reduction in local $O_{2}$ concentration, whereas the lipid tail core shows maximal enrichment of $O_{2}$. The plotted lines indicate the mean concentration for the two profiles after reaching a stable steady state, and the lighter shaded regions show the bounds within 2 standard errors of the mean.

The concentration profiles of the $O_{2}$ show that our method successfully created stable steady-state gradients across the lipid bilayer, with the profiles reflecting the amphiphilic nature of the membrane (Figure 2). In the gradient simulations, the enriched region shows elevated concentrations of $O_{2}$ when compared to the control (Figure 2, black line). Proceeding from the enriched region toward the depleted region down the *z*-axis, the $O_{2}$ concentration decreases linearly until reaching the headgroup region, where the concentration drops steeply due to the polar nature of the region. This phenomenon is observed in both gradient and control simulations as well as in other systems simulated in our lab, and has been also reported by other laboratories [29]. Entering the lipid tail core of the bilayer, the $O_{2}$ concentration increases substantially, even beyond the concentration in the bulk solution, in accordance with the hydrophobic nature of the $O_{2}$ molecule and the reported partition coefficient of $O_{2}$ [35,36]. Approaching the headgroups near the depleted region, the concentration drops steeply once again, below that of the concentration observed at the first headgroup region on the enriched side of the membrane. Exiting the headgroup region into the depleted solution, the $O_{2}$ concentration rises again. As expected, the depleted solution concentration is lower than that of the enriched side. Continuing down and away from the membrane and toward the end of the simulation box, the $O_{2}$ concentration steadily declines until reaching the biasing region at the boundary. At z=−90 Å, the concentration begins to rise due to the applied bias.

**Figure 3:**
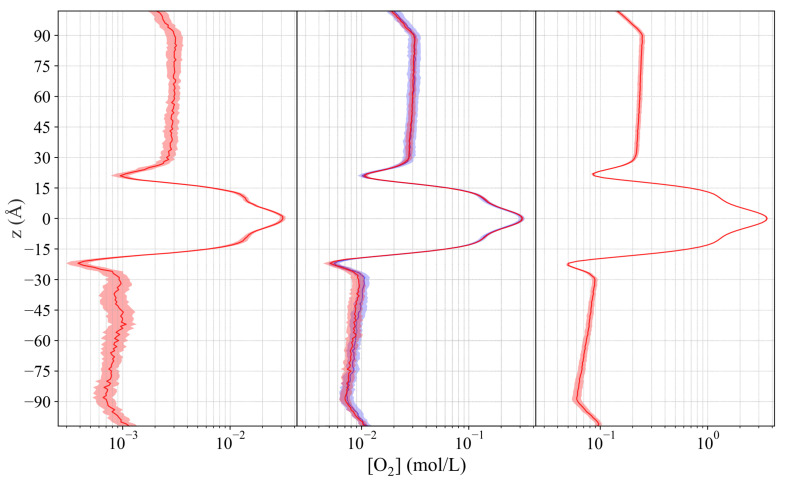
Concentration gradient profiles for the low- (5 $O_{2}$), medium- (50 $O_{2}$), and high- (500 $O_{2}$) concentration simulations ordered from left to right. The concentration profiles show the average concentration of $O_{2}$ along the z-axis, plotted as red lines with the shaded regions indicating the bounds within 2 standard errors of the mean. A concentration profile for the medium concentration system simulated using the Langevin dynamics thermostat is shown in blue, nearly identical to the simulation using CSVR. The overall features of the concentration gradients remain consistent across the various simulations.

While the absolute values of the concentrations differ between the simulations with different $O_{2}$ concentration, the gradient profiles are created similarly in all simulations (Figure 3). The limited sampling resulting from the low concentration of $O_{2}$ yields greater uncertainty for the average values in the concentration gradient profiles. A comparison of the profiles for the CSVR and Langevin dynamics simulations finds nearly identical values, showing that the choice of thermostat does not impact the energetics of the simulation (Figure 3). The relative partitioning of the $O_{2}$ molecules is consistent.

### 2.3. Oxygen Flux across the Membrane

**Figure 4:**
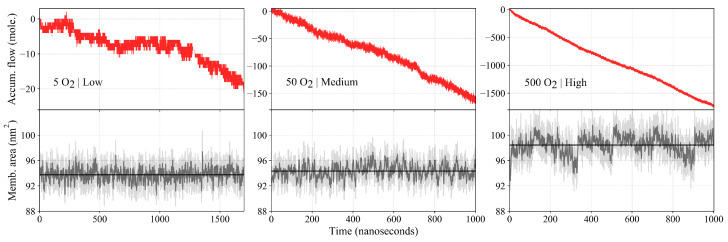
Accumulated flow (above) and membrane area (below) of the low- (5 $O_{2}$), medium- (50 $O_{2}$), and high- (500 $O_{2}$) concentration simulations. The low-concentration simulation is run for 1.7 μs to increase sampling, while the medium- and high-concentration simulations run for 1 μs. A continuous net flow of $O_{2}$ across the membrane is observed for the three simulations, and the rate of accumulated flow becomes increasingly steady and linear with increasing concentration. The low-concentration simulation exhibits substantial noise in the flow. The average flux through the membrane is found to increase with increasing the $O_{2}$ concentration, most noticeably for the high-concentration simulation.

Measuring the accumulated flow over the course of the simulation (Figure 4), we observe that the flow is proportional to the total number of $O_{2}$ molecules present in the simulation. For higher numbers of the permeant, the plot for accumulated flow becomes less noisy and approaches that of a straight line. Both the 50 and 500 $O_{2}$ simulations reach an accumulated flow of about 3.2 times the total number of permeant molecules over the course of a microsecond.

Because we measure flow at z=0, large fluctuations (“noise”) envelop the absolute value of flow which stems from the large population of $O_{2}$ in the lipid bilayer core. With an increasing number of $O_{2}$ molecules, the accumulated flow appears increasingly smooth. The low-concentration simulation visibly shows the widest fluctuations about the average. In addition to the noise, the low-concentration simulation shows a noticeable drift in the rate of accumulated flow. These two characteristics occur due to the sparse population of $O_{2}$ in the simulation such that the membrane even transiently experiences a total absence of $O_{2}$ (t≈1250ns), despite the bilayer interior providing a favorable environment for the partitioning of $O_{2}$. For the calculation of permeability, the accumulated flow is converted into a flow rate by normalizing it with respect to the block size of 100 ns.

**Figure 5:**
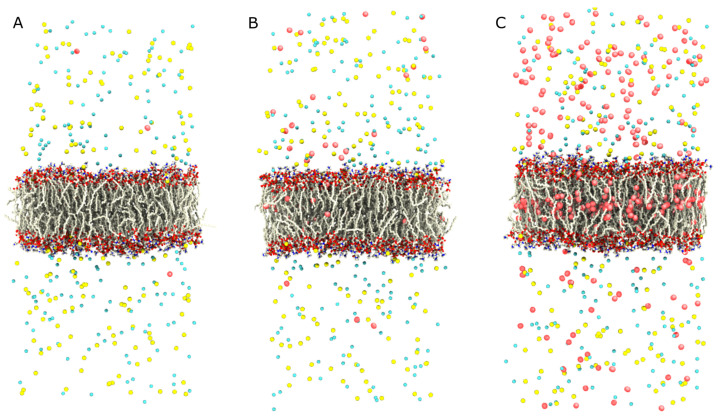
Shown are three molecular visualizations of the various concentration conditions explored by gradient simulations. Each visualization depicts a POPC bilayer at the center shown in a ball-and-stick representation. The lipid tails are colored white, while oxygen and nitrogen atoms of the head groups are colored red and blue, respectively. On either side of the bilayer is the aqueous phase which contains ${\mathrm{Na}}^{+}$ (yellow) and ${\mathrm{Cl}}^{-}$ ions colored yellow and cyan, respectively. Water molecules are not shown for clarity. Molecular oxygen ($O_{2}$), shown as two joined red spheres, is present throughout each of the systems. (**A**) Shows the “low” (5 $O_{2}$)-concentration simulation which most closely replicates a physiological concentration for dissolved $O_{2}$, although sampling was limited. (**B**) Shows the “medium” (50 $O_{2}$)-concentration simulation, representing a balanced number permeants to achieve adequate sampling for more precise measurements while minimizing the introduction of artifacts that accompany the inclusion of high permeant counts. (**C**) Shows the “high” (500 $O_{2}$)-concentration simulation, which shows a membrane whose phospholipid bilayer structure has been perturbed by the extreme and unrealistic inclusion of $O_{2}$.

The membrane area fluctuates around its average throughout the simulations (Figure 4). We find that the average membrane area increases with increasing $O_{2}$ concentration. Whereas the difference between the low- and medium-concentration simulations is slight, there is a significant increase in the membrane area for the high-concentration simulation. We attribute the increased area to the packing of a large number of $O_{2}$ molecules into the membrane interior, which cause the lipids to spread out (Figure 5).

### 2.4. Oxygen Permeability of POPC Bilayers

**Figure 6:**
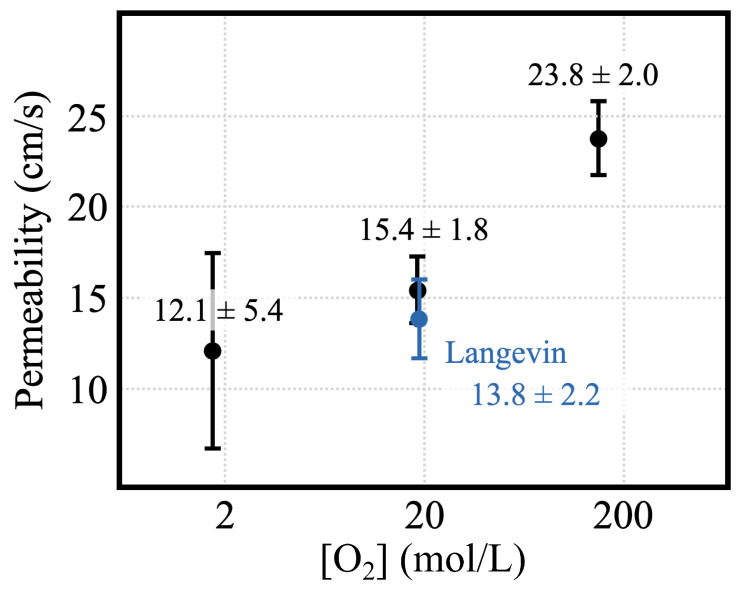
Membrane permeability displayed dependence on $O_{2}$ concentration and simulation thermostat. The calculated permeability along with standard errors are plotted in relation to the $O_{2}$ concentration revealing a correlation. Although the difference in permeability between the low- and medium-concentration simulations is not statistically significant, the downward trend appears to persist down to concentrations near 2 mM. Also plotted is the permeability from the simulation using the Langevin dynamics thermostat, which shows a slightly lower permeability from the simulation using the CSVR thermostat with an equivalent $O_{2}$ concentration.

Collating data for the concentration gradient, flow, and surface area from the simulations, we measure the permeability of POPC bilayers to $O_{2}$. We find that $O_{2}$ permeability positively correlates with the permeant concentration, measuring $P_{5}^{O_{2}}$ = 12.1 ± 5.4 cm/s, $P_{50}^{O_{2}}$ = 15.4 ± 1.8 cm/s, and $P_{500}^{O_{2}}$ = 23.8 ± 2.0 cm/s for our simulations containing 5, 50, and 500 $O_{2}$ molecules, respectively. Use of the Langevin thermostat instead of CSVR (Canonical Sampling through Velocity Rescaling) results in a reduced permeability for the simulation containing 50 $O_{2}$—$P_{50}^{O_{2},\mathrm{Langevin}}=13.8\pm 2.2$ cm/s. A summary of the simulation conditions and their results are presented in Table 1 and Figure 6, reporting the means and accompanying standard errors of pertinent measurements. We also perform control simulation for the 50 $O_{2}$ CSVR simulation, wherein no directional bias is applied. All simulations are run for 1 μs, except for the 5 $O_{2}$ simulation, which is performed for 1.7 μs. The results indicate that the permeability of the bilayer is sensitive to the permeant concentration and choice of thermostat in the simulation.

#### 2.4.1. Correlation of Permeability and Permeant Concentration

Comparing the simulations with varying levels of $O_{2}$, we find that the calculated permeability increases with $O_{2}$ concentration. While the difference is not statistically significant between the low- and medium-concentration simulations, there is a significant difference between the medium- and high-concentration simulations (Table 1 and Figure 6). The correlation exhibited between the concentration and permeability has been observed in other simulation studies. Krämer et al. [31] observed from simulations that membrane permeability to ethanol increased under elevated ethanol concentrations, with permeability calculations using either the ISD model or partial transition counting showing greater sensitivity than full permeation counting-based calculations. In another simulation study with extensive examination of concentration effects that included double bilayer simulations, Ghorbani et al. [30] found a correlation between permeability and ethanol concentration for POPC, POPE, and POPS lipid bilayers. They found that POPC showed the greatest increase, with a permeability of ∼0.10 cm/s at 1% ethanol concentration, and up to ∼1.28 cm/s at 18% when measured using the ISD model. Both discussed the breakdown of the Markovian assumption under high concentration when measuring permeability by either partial transition counting or ISD model-based methods. Although they focused their discussion on the sensitivity of methods, they touched on the fact that the presence of a solute within the membrane modulates its properties. Considering these reports and our own results, we can generalize the idea that the permeability of a membrane to a solute correlates positively with its concentration, at least for those that partition preferably and largely into the lipid phase and can change its properties.

High $O_{2}$ concentration spreads lipids, increasing permeability in simulation. Though $O_{2}$ is smaller than ethanol, aligned with its highly hydrophobic nature and large partition coefficient (∼3.5 at 37 °C) [36], we observe that most of it dissolves in the membrane and that the membrane permeability increases significantly under high, albeit unrealistic, concentration. We attribute this phenomenon to the influence of $O_{2}$ on the membrane. The high saturation of $O_{2}$ causes the membrane to expand as evidenced by the increase in membrane area resulting from the increased $O_{2}$ concentration (Table 1). The 500 $O_{2}$ simulation exhibits the greatest membrane area, markedly greater than that of the others (4.4% greater than the medium-concentration simulation). The increased area is associated with elevated permeability, although, if all else remains the same, it is expected to in fact decrease the permeability (larger area translating to reduced density of flux). However, the disruption and lipid separation caused by high O_2_ seem to produce a more “porous” membrane with higher permeability. $O_{2}$ prefers to partition into the hydrophobic lipid tail core of the bilayer, and in doing so, expands the space between lipids, importantly also in the headgroup region, making the membrane more amenable to permeating flow.

As an example, consider the ratio between the rate of flow and $O_{2}$ count. For the low-, medium-, and high-concentration simulations, the flow rate, after normalizing for the $O_{2}$ count, increases with the $O_{2}$ count, measuring 0.22/100 ns, 0.322/100 ns, and 0.343/100 ns, respectively. The increase is even more apparent when normalizing the flow rate for the solution concentrations of $O_{2}$ used to measure Δc. To explain this elevation in flow rate, and subsequently permeability, the presence of the $O_{2}$ must cause the lipids to space out and the membrane to become more leaky.

The impact of the permeant on the membrane properties raises questions about the realism of MD simulations performed at unrealistically high concentrations of the solute, which is often used to compensate for poor sampling. This is a clear example of how using high ligand counts to enhance sampling can introduce artifacts that may affect outcomes. The concentrations that we examine are high as well. With a Henry’s law constant of $H_{v}^{px}=4.7521\;\mathrm{GPa}$ at 303.14 K [37,38], we expect $O_{2}$ to be present in water at a concentration of around 250 μM under atmospheric conditions (21%). Our closest simulation system, containing only five $O_{2}$, has an average solution concentration of 1.74 mM, still an order of magnitude greater than reality. Although we find a correlation between concentration and permeability, it remains to be seen whether this effect will persist in the regime of more physiological concentrations. A confident assertion of whether the presence of $O_{2}$ continues to modulate membrane permeability at lower concentrations, which is outside the scope of the present study, would require additional simulations. It may be interesting to examine whether the positive correlation between permeability and concentration persists at more realistic concentrations of $O_{2}$ in future work. However, considering that the difference in permeability that we observe between the 5 and 50 $O_{2}$ systems is not statistically significant, the 50 $O_{2}$ simulation system seems the most practical choice, as it balances sampling and measurement precision with realism.

#### 2.4.2. Effect of Thermostat

The permeability determined from the simulations using the Langevin thermostat is lower than the simulation using the CSVR thermostat for an equivalent number of $O_{2}$ molecules. For the simulation with 50 $O_{2}$, for example, using the Langevin thermostat, we observe a permeability—$P_{50}^{O_{2},\mathrm{Langevin}}=13.8$ cm/s ± 2.2, whereas with the CSVR thermostat, the permeability is calculated to be $P_{50}^{O_{2},\mathrm{CSVR}}=15.4$ cm/s ± 1.8 (Table 1 and Figure 6).

Although the difference is not found to be statistically significant, lower permeability values resulting from the use of the Langevin thermostat have similarly been reported in the simulation literature. Dotson et al. [39] observed this effect studying $O_{2}$ when comparing Langevin to the Berendsen thermostat, as well as Kramer et al. [31] studying ethanol in comparison to Nosé–Hoover chains. Bussi et al. have reported that the CSVR thermostat preserves momentum, while the Langevin thermostat does not [40,41]. As a result, the Langevin thermostat reduces the diffusivity of particles, especially when the damping coefficient (γ) is large, when compared to NVE simulations or those using CSVR or other momentum-preserving methods. Considering the linear dependence of permeability on diffusivity in the ISD model, the reduction in permeability measured from our simulation using the Langevin thermostat is expected.

### 2.5. Dissipation of the Chemical Potential

Our concentration gradient method enables us to microscopically characterize the permeability of the membrane. The impact of concentration gradient on the dynamics may be better understood by drawing an analogy to electrical circuits. We can isolate the contribution of the concentration gradient to the relative free energy of the $O_{2}$ partitioning ΔG along the *z*-axis by comparing the concentration profile of gradient simulations to their control (Figure 2):

(3) $$\frac{{\left[O_{2}\right]}_{\mathrm{gradient}}\left(z\right)}{{\left[O_{2}\right]}_{\mathrm{control}}\left(z\right)}=e^{-\left(\Delta G_{\mathrm{gradient}}-\Delta G_{\mathrm{control}}\right)/k_{B}T}=e^{-\Delta \Delta G_{\mathrm{gradient}}/k_{B}T}$$

**Figure 7:**
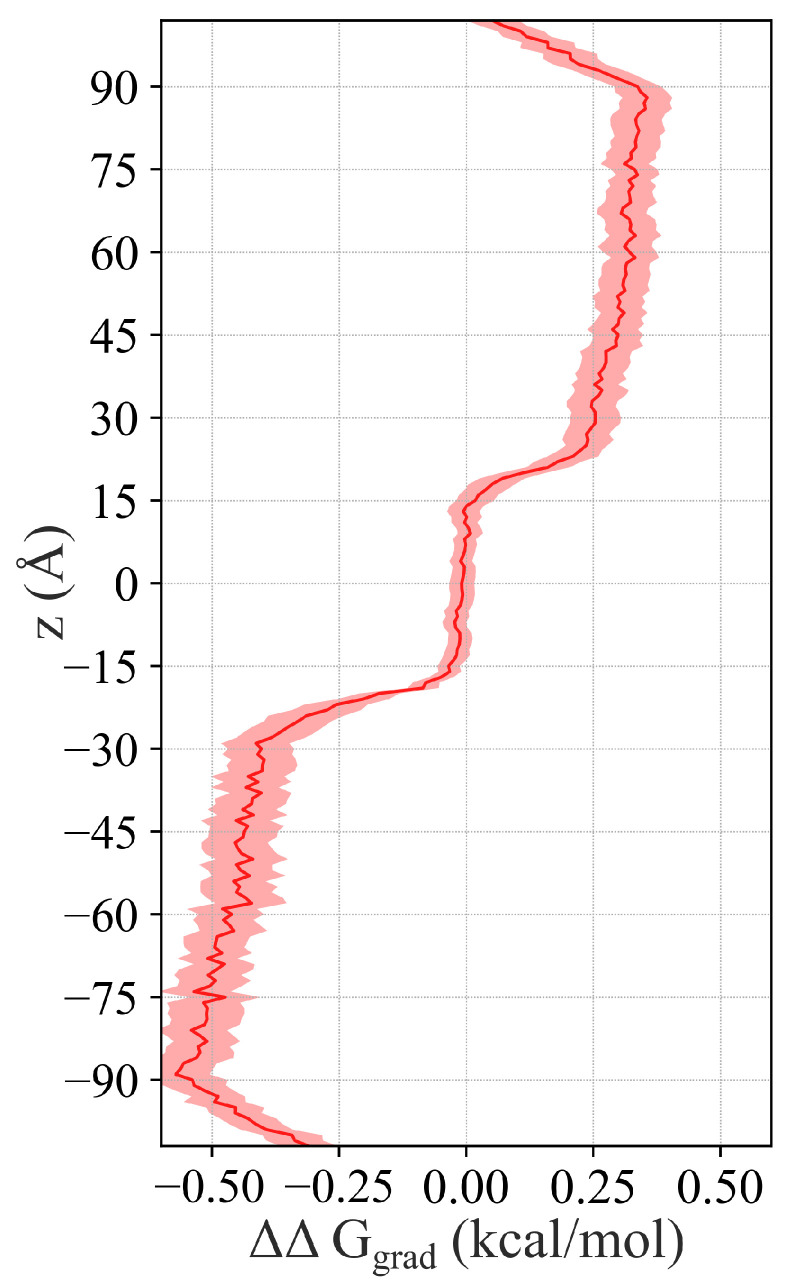
Dissipation of concentration gradient. Comparing the concentration profiles of the gradient simulation to the control enables the calculation and visualization of the gradient energy. Steep drops in the gradient, like at the lipid headgroups (z∼±18 Å), indicate regions of high resistance to permeation, whereas relatively flat regions, like within the bilayer core (z∼0 Å), indicate regions of low resistance. Viewing the energy of the concentration gradient in this way provides a precise characterization of permeability.

This calculation allows us to visualize the dissipation of the chemical potential (Figure 7). Doing so removes the enthalpic contribution to the $O_{2}$ partitioning, which originates from the interaction energy between $O_{2}$ molecules and their environment, i.e., the lipids and solution. After subtracting the enthalpy, we have access to the entropic contribution, which contains information about the organization of $O_{2}$ in the concentration gradient (loss of entropy):

(4) ΔG=ΔH−TΔS

Consider the system analogously to an electrical circuit with resistors wired in series and view the free energy through the lens of Ohm’s law:

(5) V=IR

where *V* is the voltage, *I* the current, and *R* the resistance. In our analogy, the free energy *G* can be thought of as voltage, which will decrease as the flow of $O_{2}$ (electrons), analogous to the electrical current, encounters resistance to permeation $R_{p}=\frac{1}{P}$.

Drawing an analogy to Ohm’s law enables us to determine the resistance to permeation with near-Ångstrom resolution by examining the dissipation rate of the chemical potential. Drops in the chemical potential indicate regions of high resistance, whereas a flat potential indicates negligible resistance. In this way, we can make a detailed characterization of the resistance profile of the system, which in turn determines the overall permeability. The two head group regions of the bilayer, which correspond to local minima in the $O_{2}$ concentration (z≈±25 Å in Figure 2), exhibit the greatest resistance. In fact, the majority of the energy dissipation occurs in these regions. Others have stated that only regions where the PMF rises above that of the solvent free energy will result in a decrease in permeability [29,42] since permeability depends exponentially on free energy in the ISD model. Our data support this claim. However, one could expect a permeant that encounters a rising PMF to be kinetically hindered, like at the transition from the core to the head groups, where the PMF rises steeply (10 Å <|z|<17 Å in Figure 2). Interestingly, the chemical potential does not appear to drop significantly in this region, further supporting the idea that only regions where the PMF is elevated above that of the solvent level provide resistance. There does appear to be a subtle difference between the two drops in the chemical potential at the head group regions, with the enriched side producing a smaller decrease than the depleted side. We attribute this to there being a lower concentration of $O_{2}$. Examining the dissipation, we can also characterize the solvent, which exhibits a non-zero resistance to permeability. A substantial portion of the concentration gradient dissipates within the solvent, which appears greater on the depleted side of the membrane than on the enriched side. While this is consistent with the ISD model due to the reduced $O_{2}$ concentration on this side, it is unintuitive since one would expect the permeability of the solvent to be equal on both sides. This is perhaps where we might benefit from a more general theoretical treatment of permeability, as we encounter the assumption of “small gradients” [23] in the ISD model, which does not hold in our case. The concentration of CO_2_, metabolically related to $O_{2}$, is measured to be 23 mM in arterial plasma and 11 mM within red blood cells, representing a 2:1 ratio [43]. In biological systems, the magnitude of the concentration gradient can be significant. We model a similar extrema for the concentration of a similar species of gas, $O_{2}$. In order to understand the subtleties of permeation, we may need to consider cases where the assumption of small gradients no longer holds.

### 2.6. Discrepancy between Computation and Experiments

It is commonly known that membrane permeability estimates from MD simulations differ significantly from those measured experimentally, in some cases by several orders of magnitude [11]. It is usually the case that permeability measurements via simulations are higher than those of experiments. Because of this, MD simulations have focused mostly on qualitative characterizations of permeability. Measuring the permeability of small, non-electrolytic species can prove challenging due to their rapid permeation. For example, membrane permeability to CO_2_ was measured experimentally to be on the order of $10^{-3}$ cm/s [44] for liposomes composed of *E. coli* lipids, while a similar follow-up study from the same group measured the permeability of DPPC liposomes to be on the order of $10^{-4}$ cm/s [45]. While the precision of experimental measurements of permeability may benefit from improvement, examination of our concentration gradient simulations may help to close the gap.

Significant dissipation of the concentration gradient occurs in the solvent phases over the length of a few nanometers, both in the enriched and depleted sides of the membrane. In an experiment, if the separation between the two points at which concentration is measured is large, then the solvent phase may contribute significant resistance to permeation. Points at which concentrations are measured that are far apart would yield a smaller permeability. This may very well be the case for experimental measurements of concentration since probes, like a pH sensor, may be only as close to the membrane as a few nanometers. While minuscule on the scale of experiments, a few nanometers will be in large excess of the membrane thickness, which is considered precisely in simulations. If this is the case, then experiments and simulations are almost measuring different things, not to mention the other variables in play when conducting, for example, an experiment that uses the enzymatic conversion of CO_2_ into carbonic acid and pH measurements as a readout for gas exchange across the [44]. Simulation results calculate the permeability specifically for the lipid bilayer, whereas experiments measure the lipid bilayer as well as solvent layers adjacent to it, which can contribute resistively to permeability. If the resistance to permeability of the solvent is taken into consideration, perhaps the permeability determined by experiments and the one determined in simulations can be brought closer to each other. Supporting this notion, if we calculate the permeability from our concentration gradient simulations for a slab larger than the membrane (−90Å<z<+90Å), which includes the solvent, we obtain a lower permeability of $P_{50}^{O_{2},\pm 90\;Å}=11.4$ cm/s. Möller et al. [46] suggested that the unstirred aqueous layers of around 10 nm thickness can present resistance to permeation at levels similar to that of red blood cell and EYPC (egg yolk, mostly POPC) membranes against nitric oxide, which appears a reasonable proposal in light of our data.

On the computational side, disparities may originate from inaccuracies in the classical force fields. We have shown above that even the permeability through the water phase, often dismissed perhaps as uninteresting, contributes to the overall permeability. For water, the TIP3P model, used here for the simulations, has been reported to produce a shear viscosity about twice that measured from experiments [47]. The Stokes–Einstein equation predicts that the diffusivity of a particle in solution is inversely proportional to the viscosity of the solution ($D\propto \frac{1}{\eta }$), so we can expect the diffusivity of $O_{2}$ to be overestimated by a factor of two in simulation for the water phase, translating to an overestimation of the overall permeability. In support of this idea, the self-diffusion coefficient of TIP3P water has been calculated to be approximately two times greater than experimental values (∼5.5 vs. 2.3 [$\times 10^{-9}$ $m^{2}s^{-1}$]) [48,49]. This means that we might expect the contribution to permeation resistance by the solvent to be even more substantial than observed in our simulation because the diffusivity of a solute, in our case $O_{2}$, is proportional to the self-diffusion of the solution. Like resistors in series, the water becomes a more significant contributor to the resistivity of the simulation system. The resistance to permeation of the solution phase is likely responsible for most of the discrepancy between the simulation and experiment.

Furthermore, the model for $O_{2}$ overestimates its partitioning into the lipid phase. For our work, we use the CHARMM-D, dipole model of $O_{2}$. Even though the model is among the more accurate models in terms of replicating the free energy of transfer between hexadecane and water, the energies still do not match closely: $\Delta G_{\mathrm{tr}}=6.42\;\mathrm{kcal}/\mathrm{mol}$ vs. $\Delta G_{\mathrm{tr}}=4.5\;\mathrm{kcal}/\mathrm{mol}$ experimentally [50]. It is more challenging to explain how this discrepancy will translate into changes in the calculated permeability. A reduction in partitioning into the lipid tail core of the bilayer should result in a lower permeability through the region, but it could also mean that it lowers the free energy barrier located in the headgroup region. A set of simulations comparing the results would be necessary to make a definitive statement on how the $O_{2}$ affects the modeled permeability.

## 3. Materials and Methods

### 3.1. Generating Concentration Gradients via a Unidirectional Bias at the Boundary

We generate concentration gradients in the MD simulation by applying a unidirectional bias to the motion of select particles near the periodic boundaries. Such a bias drives the select particles across the periodic boundary into the next periodic image in one direction (Figure 1). This causes a local reduction in the concentration of the particles at one end (depletion) while raising it at the other (enrichment). Spatially restricting the application of the bias to the simulation boundary allows the select particles to diffuse freely within the rest of the simulation volume (the core) and naturally establish a concentration gradient. The absence of bias from the core is an important design goal of our method, as it allows users to observe the stochastic movement of permeants in a region of interest, for example, across the membrane.

We achieve the directional bias using Grid-steered molecular dynamics [51] (G-SMD or “grid-forces”), a module in NAMD [52,53] that allows the user to impose, on select particles, an external force derived from the gradient of a user-defined scalar field (grid potential). We define the (grid) potential to be flat within the core of the simulation box so as not to interfere with the natural dynamics of particles in this region. The grid potential extension to the boundaries along the long axis experiences a fixed energy step between periodic images, thus creating a linear energy ramp that biases the motion of select particles between periodic images. The resulting effect is that the concentration of the select particles at opposite sides of the boundary experiences a gradient proportional to the magnitude of the imposed energy step between periodic images.

### 3.2. POPC Bilayers for Concentration Gradient Simulations

Testing our concentration gradient method as a means to accurately characterize permeability, we apply it to $O_{2}$ in μs-scale MD simulations of POPC membranes. We simulate a 100 Å × 100 Å patch of POPC, generated using CHARMM-GUI [54], solvated on both sides to a total height of 220 Å. We define an unbiased region that stretched from z=−90 Å to z=+90 Å, and impose a 1 kcal/mol energy step between periodic images of the simulation on the $O_{2}$ (Figure 1). We perform a set of simulations with varying $O_{2}$ concentration to observe the effect of permeant concentration on permeability, populating each simulation system with either 5, 50, or 500 $O_{2}$ molecules by randomly selecting and replacing water molecules. Our simulations primarily use the canonical sampling through velocity re-scaling (CSVR) thermostat [40]. In addition, we also perform a simulation using the Langevin thermostat to compare the impact of the thermostat choice. To perform the permeability analysis, we characterize the concentration gradient across the system and measure the flux of $O_{2}$.

### 3.3. Molecular Dynamics Technical Specifications

The lipid bilayer in the membrane simulation systems contains 294 POPC molecules-The membranes are solvated in TIP3P water using the SOLVATE plugin of VMD [55], with the solvent box size measuring 100 Å × 100 Å × 220 Å in the *x*, *y*, and *z* dimensions, respectively. The AUTOIONIZE plugin, also included with VMD, is used to add ${\mathrm{Na}}^{+}$ and ${\mathrm{Cl}}^{-}$ ions to create a 150 mM salt solution, emulating a biological solvent. The solvent consists of 56.5k water molecules with 159 ${\mathrm{Na}}^{+}$ and 159 ${\mathrm{Cl}}^{-}$ ions. $O_{2}$ molecules are introduced by random selection and replacement of water molecules. The total size of the system is 209k atoms. Our simulations use the CHARMM36m force field [56,57] and the CHARMM-D, dipole model for $O_{2}$ ($\epsilon =-0.12\;\mathrm{kcal}/\mathrm{mol};R_{min}/2=1.7\;Å$; $l_{bond}=1.23\;Å$; q=±0.021e) [58,59,60]. Simulations are performed with NAMD2.14 [52,53], first equilibrating for 15 ns and then performing production runs for 1 μs, except for the 5 $O_{2}$ system, which is run for 1.7 μs. To prevent a drift in the lipid bilayer with relation to the applied gradient, the center of mass of the lipids is restrained to z=0 using a harmonic restraint with a force constant of 1 kcal/mol using the collective variables [61] module in NAMD.

### 3.4. Characterization of Concentration Gradients

We profile the concentration gradient of $O_{2}$ in the simulations by measuring its local concentrations in thin 1-Å slabs along the simulation *z*-axis (membrane normal). The local concentrations are measured for each saved frame of the simulation trajectory to obtain a time average. The concentration profiles of the gradient simulations show elevated concentrations on the enriched side of the membrane, and a decreased concentration on the depleted side when compared to the control simulation (Figure 2). The profiles also highlight the amphiphilic features of the POPC lipid bilayer membrane.

Measuring permeability using the relation derived from Fick’s first law (Equation (1)) requires the characterization of Δc. Using the concentration profiles to inform our selection, we choose the difference in concentration at regions just outside the lipid headgroups, within a few Ångstrom, at z=±30 Å,

(6) Δc=c(z=+30Å)−c(z=−30Å).

$O_{2}$ concentrations begin to drop just after ±30 Å points before entering the lipid bilayer. These bounds precisely indicate the full extent of the membrane’s influence on the $O_{2}$, and the point of complete transition into the solution phase. Our observation agrees with X-ray and neutron scattering experiments showing that the atomic density of a POPC bilayer becomes 0 at 30 Å [62].

### 3.5. Flux Calculation

Returning to measuring permeability, we measure flux *J*, which is defined as the rate of flow per unit area. From our simulations, we calculate flux by dividing the accumulated flow of $O_{2}$ through the membrane over time Δt by the average membrane area *A*:

(7) $$J=\frac{Accumulated\;flow}{Area\times \Delta t}$$

**Figure 8:**
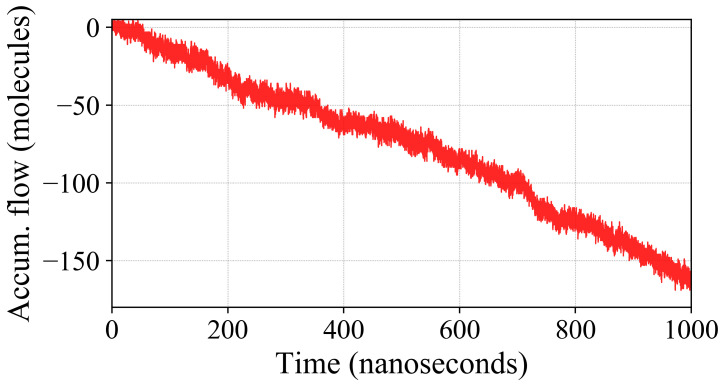
A sustained concentration gradient was induced, causing our simulations to experience a net flow of permeants through the system over time. By tracking the accumulated flow of permeants, we can determine the average rate of flow. Shown is a plot for the accumulated flow of a simulation with 50 $O_{2}$. There is a relatively linear accumulation of $O_{2}$ over the course of the 1 μs simulation. Because flow is measured across the z=0 plane, there is substantial noise from the repeated up and down crossings in this high-concentration region of the membrane, but the net flow is in the direction of the applied gradient (negative *z*).

We define the accumulated flow as the total number of crossings of the permeant, $O_{2}$, across a boundary (e.g., the membrane) over the course of a simulation (Δt). And due to conservation of mass in the time-average steady state,

(8) $$\frac{J(z)}{dz}-\frac{c(z)}{dt}=0$$

we assume the flow to be consistent along the length of the simulation box, only requiring that it be measured across one boundary. The accumulated flow (Figure 8) was measured by analyzing the simulation trajectories in VMD [55] using in-house Tcl scripts, described in the following text.

Two characteristics of MD simulations must be considered when determining flow. First, MD simulations are discrete in both time and space, especially in the context of saved trajectories, where instantaneous snapshots of simulations are saved at regularly spaced intervals. Second, MD simulations typically use periodic boundary conditions (PBCs) to reduce edge effects, which introduce a major discontinuity to the position of particles when they traverse a boundary. Because of these two conditions, care must be taken when determining flow.

**Figure 9:**
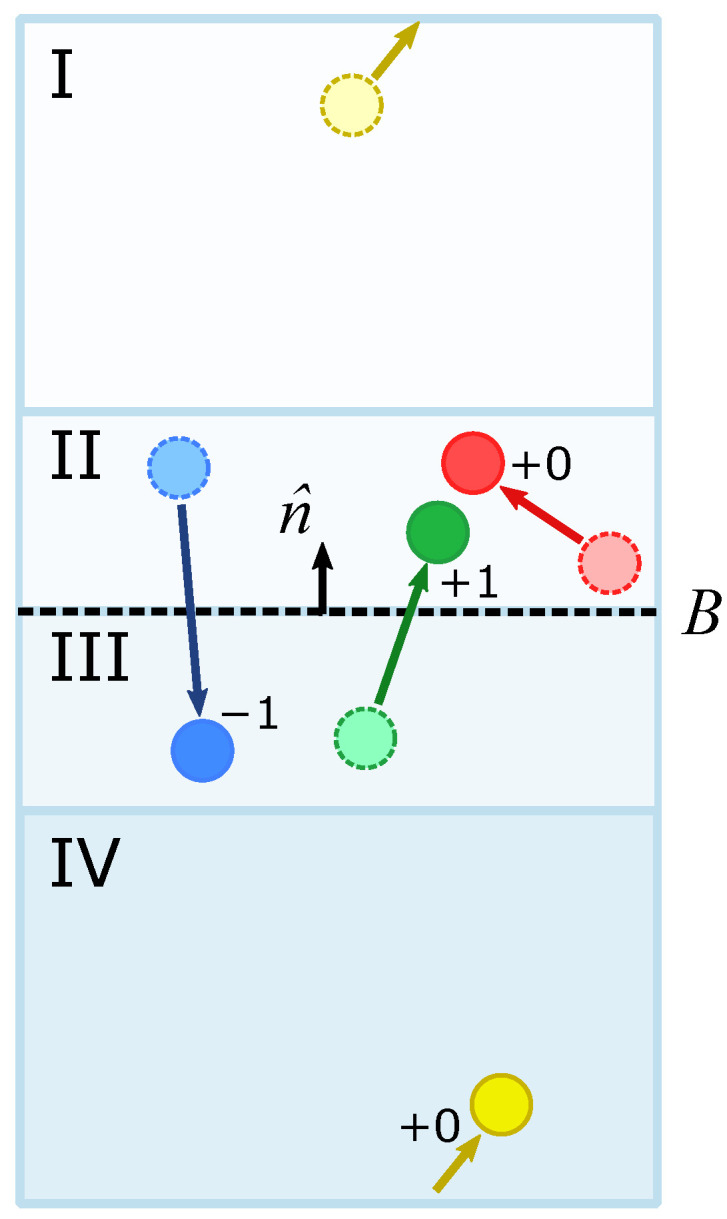
A flow counting algorithm was implemented to track the flow of particles across a boundary, *B*, with surface normal $\hat{n}$. The algorithm for counting divides the area of interest into four regions, I, II, III, and IV, and tracks the movement of particles between them by comparing frames of the trajectory. Particles that move across the boundary in the same direction as $\hat{n}$ (III to II, green particle) contribute +1 to the flow count. Conversely, particles that move across *B* against $\hat{n}$ (III to II, blue) contribute −1 to the flow. Particles that remain in the same compartment contribute 0 to flow (II, red). To avoid erroneous flow counts that might arise from a particle crossing the periodic boundary, particles that move between the flank regions also contribute 0 to flow (I to IV, yellow).

We define four regions in the simulation space, which we shall call regions I, II, III, and IV (Figure 9). The boundary across which we would like to determine flow, *B*, divides regions II and III. We also define a normal vector $\hat{n}$ for *B* that points in the direction from region III to region II. Now, for each saved frame in the simulation trajectory, particles are labeled according to the region they reside in. The positions of the particles are then compared between the consecutive frames. If a particle moves from region III to region II, we consider it to contribute +1 to the flow, in accordance with $\hat{n}$. If a particle moves from region II to region III, we consider it to contribute −1 to the flow. Movements between the flanking regions across the periodic boundaries, i.e., between regions I and IV, are ignored.

If the length of the simulation time between the trajectory frames is too long, it might be possible for a permeant particle to also traverse the periodic boundary and then the traverse therefore not to be counted. Also, if a particle moves from region I to III from one frame to another, there will be zero contribution to the flow. Paying attention to the sizing of the four regions helps avoid some of these problems. In this study *B* represents the bilayer, and regions II and III each have a length of 40 Å placed on either side of the lipid bilayer, while regions I and IV occupy the remaining length (∼70 Å) of the simulation box. Considering that the diffusivity of oxygen is reported to be approximately 8 $\times 10^{-5}$ ${\mathrm{cm}}^{2}$/s = 8 ${Å}^{2}$/10 ps [29], and frames are saved every 10 ps, the region lengths are deemed to be of sufficient length.

**Figure 10:**
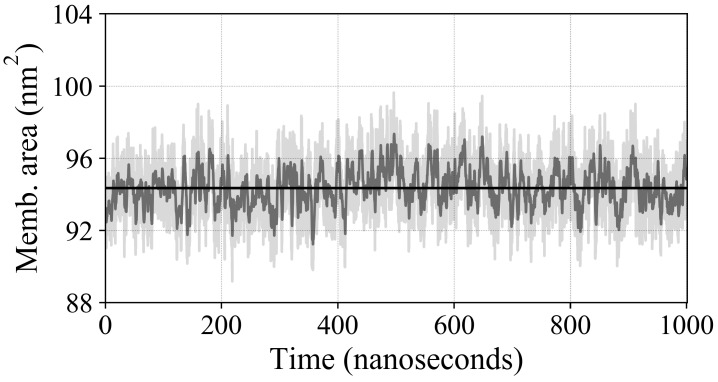
Membrane area for 50 $O_{2}$ simulation. The instantaneous, exponentially weighted moving average, and whole simulation average of the membrane area over the course of 1-μs are plotted in light gray, gray, and black, respectively. The flow rate of $O_{2}$ is normalized using the average area to yield the flux.

To measure area, we query our simulation data for the lengths of the lateral xy dimensions. Each of these measurements is recorded and contributes to the average membrane area for our flux calculations (Figure 10).

### 3.6. Calculating Membrane Permeability

We determine the permeability of the POPC bilayers by combining gradient, flow rate, and surface area, averaged over 100 ns blocks from our simulations:

(9) $$P=\frac{J}{\Delta c}=\frac{Accumulated\;flow}{Area\times \Delta t\times \Delta c}$$

where Δt is the block size used for block averaging. The concentrations at z=±30 Å are used to calculate the gradient. Incorporating Δt with accumulated flow and area is necessary to transform our flow into a flow rate.

### 3.7. Error Analysis via Block Averaging

The positions of particles are highly autocorrelated in MD simulations, especially when frames are saved with short intervals. This autocorrelation disallows the simple use of the number of frames as the number of samples *N* to estimate standard errors (SEs). Estimating error as $SE\approx \frac{\sigma }{\sqrt{N}}$ invokes the underlying central limit theorem, which assumes that each sample is independent. We do not assume independence for each trajectory frame, as they are saved every 10 ps. Doing so would erroneously underestimate the statistical error and subsequently overestimate the precision. To obtain a more accurate quantitation of error, we divide our simulations into 100 ns blocks and calculated the mean values for the properties of interest, treating them as independent samples—block averaging [63].

## 4. Conclusions

In this paper, we introduce a non-equilibrium method to create a steady-state concentration gradient in MD simulations and demonstrate its utility in measuring the permeability of membranes. Because the bias used to generate the gradient is isolated to only a small region at the boundaries of the simulation box, the motion of affected permeants remains diffusive, Brownian, in the region of interest, namely, the membrane. The free flow of permeants through the system and across the membrane can then be measured to characterize permeability in a manner consistent with experiments. We have shown that the resulting permeability measurements agree with previously reported values for an exemplary system consisting of $O_{2}$ and a POPC bilayer, while also reporting the sensitivity of calculated permeability to the choice of thermostat and permeant concentration. Because this method calculates the bulk permeability of the membrane as opposed to along a single path as is the case when using the ISD-model, it can be applied to a wide variety of permeants and membranes of complex, heterogeneous composition and structure, including even proteins. In addition, the method allows the user to readily pre-determine the magnitude of the concentration gradient by modulating the quantity of the permeant and imposed gradient potential to emulate the experimental conditions. Lastly, the users can measure the dissipation of the concentration gradient potential along the system to identify regions of high resistance to permeation.

## 5. Future Work

This report sets the foundation for future work on the examination of simulation artifacts and complex biological membrane systems. We observe a relationship between the total permeant concentration and the permeability of the membrane, which we hypothetically attribute to the membrane modulating effect of the $O_{2}$. In simulations, we usually tend to enhance sampling by employing high ligand concentration, which may introduce artifacts that affect the simulation properties. We observe that artificially high concentrations produce an increase in permeability. It may be interesting to examine whether the positive correlation between permeability and concentration is eliminated for more realistic $O_{2}$ concentrations. Additionally, we would like to examine more complex membranes composed of heterogeneous lipids types and even containing proteins, which represent more closely biological membranes, which consist of integral and peripherally associated components. 

## Figures and Tables

**Table 1:** Summary of data used for permeability calculations. Tabulated are the simulation conditions and collected data used to calculate the $O_{2}$ permeability of a POPC bilayer. The average values are reported along with standard errors of means.

$O_{2}$ Count	Length (μs)	Thermostat	${\left[O_{2}\right]}_{z=+30\;Å}$ (mmol/L)	${\left[O_{2}\right]}_{z=-30\;Å}$ (mmol/L)	Flow Rate (molecules/100 ns)	Area (${Å}^{2}$)	Permeability (cm/s)
5	1.7	CSVR	2.56 ± 0.19	0.92 ± 0.12	1.12 ± 0.48	9375 ± 5	12.07 ± 5.41
50	1.0	CSVR	27.77 ± 1.07	9.38 ± 0.62	16.1 ± 1.58	9434 ± 10	15.41 ± 1.84
500	1.0	CSVR	210.35 ± 6.00	88.67 ± 3.21	171.4 ± 10.88	9847 ± 25	23.75 ± 2.01
50	1.0	Langevin	27.38 ± 1.63	10.52 ± 0.61	13.2 ± 1.55	9399 ± 12	13.84 ± 2.16

## Data Availability

Molecular dynamics simulation system data (PDB & PSF files) are available on Zenodo (https://zenodo.org/doi/10.5281/zenodo.10654888, accessed on 13 February 2024), along with exemplary configuration files and final snapshots of simulation trajectories. Additional data can be made available upon request.

## Abbreviations

The following abbreviations are used in this manuscript:

CSVR	Canonical Sampling through Velocity Rescaling
G-SMD	Grid-Steered Molecular Dynamics
ISD	Inhomogeneous Solubility Diffusion
MD	Molecular Dynamics
PBC	Periodic Boundary Conditions
PMF	Potential of Mean Force
